# Modeling social interaction dynamics measured with smartphone sensors: An ambulatory assessment study on social interactions and loneliness

**DOI:** 10.1177/02654075221122069

**Published:** 2022-08-20

**Authors:** Timon Elmer, Gerine Lodder

**Affiliations:** 1 Department of Psychometrics and Statistics, Faculty of Social and Behavioural Sciences, 3647 University of Groningen, Groningen, The Netherlands; 2 Department of Humanities, Social and Political Sciences, 27219 ETH Zürich, Zürich, Switzerland; 3 Department of Developmental Psychology, Tilburg School of Social and Behavioral Sciences, 120694 Tilburg University, Tilburg, The Netherlands

**Keywords:** Loneliness, social interaction, ambulatory assessment, social sensors, passive sensing, digital phenotyping

## Abstract

More and more data are being collected using combined active (e.g., surveys) and passive (e.g., smartphone sensors) ambulatory assessment methods. Fine-grained temporal data, such as smartphone sensor data, allow gaining new insights into the dynamics of social interactions in day-to-day life and how these are associated with psychosocial phenomena – such as loneliness. So far, however, smartphone sensor data have often been aggregated over time, thus, not doing justice to the fine-grained temporality of these data. In this article, we demonstrate how time-stamped sensor data of social interactions can be modeled with multistate survival models. We examine how loneliness is associated with (a) the time between social interaction (i.e., interaction rate) and (b) the duration of social interactions in a student population (N_participants_ = 45, N_observations_ = 74,645). Before a 10-week ambulatory assessment phase, participants completed the UCLA loneliness scale, covering subscales on intimate, relational, and collective loneliness. Results from the multistate survival models indicated that loneliness subscales were not significantly associated with differences in social interaction rate and duration – only relational loneliness predicted shorter social interaction encounters. These findings illustrate how the combination of new measurement and modeling methods can advance knowledge on social interaction dynamics in daily life settings and how they relate to psychosocial phenomena such as loneliness.

## Introduction

New technological advancements allow researchers to capture individuals’ experiences at a fine-grained level as they go about their day-to-day lives (Miller, 2012). Assessments in daily life settings, so-called ambulatory assessments (Trull & Ebner-Priemer, 2014), have the key advantage over more traditional lab-based or survey-based methods in that they allow researchers to study participants’ experiences in natural settings and their moment-to-moment changes.

In particular, the study of social interactions has profited from measurements in daily life settings, as the fine-grained temporal level matches the timescale in which social interactions happen. Ambulatory assessed data on social interactions have provided insights into numerous psychosocial phenomena (e.g., Elmer & Stadtfeld, 2020; Quoidbach et al., 2019; Vogel et al., 2017). For example, Mehl et al. (2010) showed that well-being was associated with spending less time alone and more time in conversations (also see Milek et al., 2018).

The development of research questions and theories on causes and consequences of social interactions in daily life has gone hand-in-hand with new technological advancements in measuring the complete set of social interactions that individuals experience in their day-to-day life. For this, scholars have used a variety of methods, ranging from event-contingent experience sampling (e.g., Reis & Wheeler, 1991), diary methods (e.g., Gochmann et al., 2022), electronically activated recorders (Mehl et al., 2001), to sensors worn as a nametag (Elmer et al., 2019). Recently, also the measurement of social interactions via a participant’s smartphone has been proposed – with the key advantage that these assessments do not need any active input from the participant, which provides a low burden for participants and, as such, allows researchers to record interactions over more extended periods of time (Bachmann, 2015; Fulford et al., 2021). So far, however, sensor data have often been aggregated over time (e.g., Elmer & Stadtfeld, 2020; Milek et al., 2018), thereby not doing justice to the fine-grained temporality of these data. Thus, as of yet, it has not yet been demonstrated how fine-grained smartphone-sensor data on social interaction dynamics can be modeled and how this granularity allows gaining new insights into the relationship between social interactions and psychosocial phenomena, such as loneliness. This article aims to fill this gap.

In the remainder of this section, we first describe the theoretical relationships between social interactions and loneliness that motivated our exemplary research questions. Second, we introduce the current study and its unique multi-method design, including smartphone sensors.

### The relationship between social interactions and loneliness

The feeling of loneliness arises when individuals experience a discrepancy between the desired and actual quantity or quality of social interactions (Peplau & Perlman, 1982). Prolonged feelings of loneliness can have serious consequences for mental and physical health: Loneliness is associated with numerous mental and physical illnesses, which contributes to a lower life expectancy (S. Cacioppo et al., 2015; Holt-Lunstad et al., 2015). The feeling of loneliness is, by definition, intertwined with our experience of social relationships. Existing research suggests that broader measures of social relationships, such as the quality of friendship and the number of friends one has, are relevant for the development of loneliness (Schwartz-Mette et al., 2020). Yet, much less is known about the day-to-day social experiences related to loneliness, such as social interactions and solitude^1^ (van Roekel et al., 2016). The use of (smartphone) sensors to capture social interaction in daily life settings, therefore, has the potential to advance our understanding of the relationship between social interactions and loneliness.

Social interactions are essential for forming stable social relationships and are thus, over time, related to trait loneliness (Schwartz-Mette et al., 2020). Yet, social interactions and the feeling of loneliness are also more directly intertwined: The loneliness model by Cacioppo and Hawkley suggests that when basic human needs of social interactions are thwarted, individuals start to feel lonely as a result of this threat (J. T. Cacioppo et al., 2006; J. T. Cacioppo & Hawkley, 2009; Hawkley & Cacioppo, 2010). Thus, loneliness can result from the subjective evaluation of the fit between the expected and the actual number of social interactions (De Jong Gierveld et al., 2016; Peplau & Perlman, 1982). Importantly, loneliness cannot only be the result of (a lack of) social interactions, but loneliness can also affect how individuals engage in social interactions (Qualter et al., 2015).

On the level of day-to-day social interactions, two mechanisms of how loneliness affect social interactions have been proposed: On the one hand, loneliness could result in a motivation to reconnect and thus predict more or longer social interactions, but on the other hand, loneliness could result in withdrawal and thus predict fewer or shorter interactions (Qualter et al., 2015). As a consequence of such behavioral tendencies of state loneliness, individuals who do not frequently interact with others may fail to build stable social relationships – thus contributing to higher trait loneliness (Schwartz-Mette et al., 2020).

Thus, based on current findings, it is unclear whether loneliness mainly results in increases in interactions or withdrawal from interactions. Moreover, as existing findings are primarily based on participants’ self-reports, effects may be overestimated because of common method biases (Podsakoff et al., 2003). We thus investigate if loneliness predicts social interactions measured with automatically sensed social interaction data.

We differentiate between joining and leaving social interactions as these are two processes with different underlying social mechanisms (Hoffman et al., 2019). Differentiating between joining and leaving social interactions can help to determine, for example, whether loneliness is more associated with the initiating of social interactions (i.e., joining process or the rate of interactions) or with leaving interactions once they are established (i.e., leaving process or the duration of interactions). Thus, we examine whether loneliness is associated with (a) the time between social interactions (i.e., interaction rate; Research Question 1 [RQ1]) and (b) the duration of social interactions (RQ2)?

### The present study

To investigate our research questions, we used data from a student population in which loneliness was measured before a 10-week ambulatory assessment phase (Wang et al., 2014). During the ambulatory assessment phase, the StudentLife sensing application on students' smartphones measured when the students were in a social interaction (*N*_*obs*_ = 74,645). The assessment of psychological data via smartphone sensors is often referred to as digital phenotyping, which is the “moment-by-moment quantification of the individual-level human phenotype in-situ using data from smartphones and other personal digital devices” (Torous et al., 2016, p. 2). The StudentLife smartphone application, assessing phenomena with a range of sensors, such as GPS and microphone (Wang et al., 2014), is unique among the set of sensing applications, as it assesses digital phenotypes of sociability together with survey-based measures of loneliness. For a comparison of digital phenotyping apps in mental health research, see Mendes et al. (2022). This study design is unique in two ways, allowing us to gain new insights into the interplay between the dynamics of social interactions and feelings of loneliness.

First, the combination of traditional survey methods (assessing loneliness) with state-of-the-art passive sensing technologies (measuring social interactions) provides a multi-methods setting, where common-method biases are reduced (Goossens & Beyers, 2002). The time-stamped nature of the smartphone sensor data allows us to differentiate processes of joining and leaving social interactions (RQ1 and RQ2). These processes can be modeled with multistate survival models, which we introduce in more detail in the Methods section. Multistate survival models have rarely been applied within psychological research, despite their importance for modeling the timing of mutually exclusive states – such as being in an interaction and being alone (Stoolmiller & Snyder, 2006). This article demonstrates how multistate survival models can be applied to model the dynamics of time-stamped social interaction data.

Second, we consider that there are different types of loneliness relating to different spheres of social interactions (S. Cacioppo et al., 2015; Luhmann et al., 2016). Some individuals feel lonely because they seek a close and intimate attachment (*intimate* *loneliness*; S. Cacioppo et al., 2015). Other sources of loneliness can come from a feeling of lacking social relationships and a lacking of belonging in different social networks such as family networks, friendship networks, or classroom networks (*relational* *loneliness*; Maes et al., 2019). The third type of loneliness comes from a feeling of lacking connections to similar others in society (*collective* *loneliness*; S. Cacioppo et al., 2015). In this study, we differentiate between these three types of loneliness: intimate, relational, and collective loneliness.

Taken together, in this study, we explore relationships between loneliness and (a) the time between interactions (i.e., interaction rate), and (b) the duration of social interaction in a student sample, using novel measurement and analysis methods. To test the robustness of our findings, we report additional analyses, including effects of smartphone usage, extraversion, and the overall level of loneliness on social interaction dynamics. All data and analysis scripts can be accessed at osf.io/c94vs

## Materials and methods

### Participants

Forty-eight students of a computer science program participated in the Dartmouth StudentLife study (Wang et al., 2014). The data of this study are publicly available (studentlife.cs.dartmouth.edu). Unfortunately, no information about participants’ gender and age was made available in the dataset. The data owners were also not willing to share this information upon request. However, in the data-introduction paper, Wang et al. (2014) report that the sample was predominantly male (38 participants, 79%). Most students were undergraduate students (63%). Most participants identified as either Caucasian (*N* = 23, 48%) or Asian (*N* = 23, 48%). Two participants (4%) identified as African-American. The data collection was approved by the Institutional Review Board at Dartmouth College.

Participants were recruited through a computer science class at Dartmouth College in 2013. All 75 participants of the class were invited. Sixty participants agreed to participate in the study. Twelve students decided to drop the class (*N* = 5) or the study participation (*N* = 7). The remaining 48 students participated in the ambulatory assessment part. Of those, 45 participants filled out the T1 loneliness survey. Our analyses on predicting social interaction dynamics from loneliness (RQ1, RQ2), thus were carried out with the sample of 45 participants.

### Procedure

The study consisted of online surveys and a 10-week ambulatory assessment phase covering the entire duration of one university term at Dartmouth (United States). Students were incentivized with two prize draws at the end of weeks three and six to win technical devices such as smartphones.

During the online survey that was administered before the ambulatory assessment phase, participants reported on various mental health dimensions (e.g., perceived stress, depressive symptoms, loneliness) and Big Five personality traits (John & Srivastava, 1999).

During the ambulatory assessment phase, participants were asked to carry a smartphone with the StudentLife sensing app. Students that did not have a smartphone were offered to use a Nexus 4s smartphone during the study. Each student received a one-to-one tutorial on what the application does and how to use it. The StudentLife app had two purposes: (1) Prompting participants with experience sampling questionnaires and (2) passively sensing participants’ behavior. These experience sampling data were not used in this study because participants did not fill them out regularly. The StudentLife sensing app measured participants’ physical movement through GPS and accelerometer sensors (for more details, see Wang et al., 2014). Relevant to this study is the automated classification of conversation data through the smartphone’s microphone, which provides information about the start and the end time of participants’ conversation periods. From this data, the number of interactions and their duration can be derived.

### Measures

#### Social Interactions

Interactions can be defined as “reciprocal influence of individuals upon one another’s actions when in one another’s immediate physical presence” (Goffman, 1956, p. 18). In the present study, we used automatically recorded conversations as indicators of interactions; thereby, we are confident that there is an exchange between people and that these people are in each other’s immediate physical presence.

Using the input from the smartphone’s microphone, a machine learning algorithm was applied to classify the audio signal into periods in which participants were part of a conversation. The sensing algorithm ran locally on the smartphone so that no audio data had to be transmitted to the researchers. The algorithm was shown to validly and reliably detect social interactions in prior studies (Lane et al., 2011; Rabbi et al., 2011). For instance, Lane et al. (2011) compared self-reports of interactions with those obtained through the smartphone’s microphone and the sensing algorithm. They concluded that because of the algorithm’s sensitivity towards classifying audio snippets that are “ambient sound from activities that are not actual conversations (e.g., when the user is watching TV)” (p. 7) as interactions, 14% of the detected interactions were not reported in the self-reports. Evaluations of similar detection algorithms show a comparably high accuracy, with up to 92% correctly identified interactions (Feese & Tröster, 2013). Conversation information during lectures and class meetings was removed from the dataset (Wang et al., 2014).

#### Loneliness

Loneliness and its three subscales (intimate, relational, and collective loneliness) were measured with the Revised UCLA Loneliness Scale (Russell, 1996). The scale consists of 20 items assessing how often the participant felt the way described in the item. Subscales for intimate, relational, and collective loneliness were calculated in line with Luhmann et al. (2016). Intimate loneliness was measured with three items such as “there are people I feel close to” (α = .87), relational loneliness with three items such as “there are people I can talk to” (α = .67), and collective loneliness with three items such as “I have a lot in common with the people around me” (α = .78). Cronbach’s α for the entire scale (omitting the subscales), as used in sensitivity analyses, was .93. The average loneliness score was 1.96 (*SD* = 0.58).

#### Control variables

##### Smartphone usage

During the ambulatory assessment phase, the smartphone also collected data on its use (i.e., when the screen was unlocked). To assess the association between short-term phone usage and the rate and duration of social interactions, we coded a dummy variable as one when the participant was using the smartphone within the last hour before the start of a social interaction event, and zero otherwise. On average, participants were using their smartphones in 74.5% (*SD* = 10.4%) within the hour before the start of a social interaction event.

##### Extraversion

Prior to the ambulatory assessment phase, participants responded to the Big Five Inventory (John & Srivastava, 1999), which assesses the Big Five personality traits. The Extraversion subscale consists of eight items such as “I see myself as someone who is talkative”, measured on a five-point scale ranging from “Disagree strongly” (1) to “Agree strongly” (5). The average of these items constitutes the Extraversion score, which in this sample was 2.98 (*SD* = 0.72). The Extraversion score is only used in the sensitivity analyses reported in the Supplementary Materials.

### Analytical strategy

#### Is loneliness associated with (a) the time between social interactions (i.e., interaction rate; RQ1) and (b) the duration of social interactions (RQ2)?

To model the effects of loneliness on the dynamics of social interactions, we use multistate models (Stoolmiller & Snyder, 2006). Multistate models are based on Cox’s proportional hazard model (Cox, 1972), which estimates how independent variables are associated with the probability of observing a *single type of event* at a particular point in time (e.g., social interaction event, illness event).

In multistate models, the transition times between *two or more* mutually exclusive states are modeled. In our data, participants transition between “alone” and “interaction” states, as illustrated in the data excerpt shown in Table 1. Table 1 shows the first six observations for the individual with ID = 1. The start and end columns indicate from when to when that individual was in a particular state (see state column). In our case, the transition times from being in an “alone” state to an “interaction” state (joining social interactions) and the transition times from being in an “interaction” state to an “alone” state (leaving social interactions) are being modeled. Put simply, the dependent variable of the multistate analysis is the time until a transition is observed (see the last column in Table 1).

**Table 1.** Exemplary data excerpt.

ID	State	Start	End	Time in state [in s]
1	alone	27.03.13 07:17:45	27.03.13 07:25:16	451
1	interaction	27.03.13 07:25:16	27.03.13 07:49:53	1477
1	alone	27.03.13 07:49:53	27.03.13 07:50:33	40
1	interaction	27.03.13 07:50:33	27.03.13 07:51:34	61
1	alone	27.03.13 07:51:34	27.03.13 08:03:06	692
1	interaction	27.03.13 08:03:06	27.03.13 08:04:26	80

By estimating the relative risk (i.e., hazard ratio) of a covariate (e.g., loneliness) on individuals' tendencies to change to another state, we can assess how loneliness is related to joining and leaving social interactions. In addition, we can take the participant’s past social behavior and temporal dynamics into account (e.g., changes in fluctuations in the structure of interactions governed by the time of the day). Multistate models also take into account that observations are nested within individuals by estimating robust standard errors (Putter et al., 2007). For a detailed mathematical introduction to multistate models, see Putter et al. (2007).

Although the application of multistate models is common in neighboring disciplines such as medicine and sociology (e.g., Bijwaard, 2014; Putter et al., 2007), they have rarely been applied within psychological research. One of the few exceptions is the analysis by Stoolmiller and Snyder (2006), who study emotional expression within child-parent interactions in a laboratory setting.

To test RQ1, we add the three loneliness subscales measured at T1 as predictors of transitions from the alone to the interaction state (rate) and from the interaction to the alone state (duration). Further covariates, controlling for general social interaction dynamics, consist of dummy variables for the time of the day (night, morning, afternoon, evening), whether or not the interaction took place on the weekend (dummy variable with 0 = weekday, 1 = weekend), window variables capturing the number of social interactions in the past two and 24 hr, and the mean duration of social interactions in the past 2 hr. These variables account for tendencies of interactions to occur at a higher rate during the daytime (than at night) and for the influence of previous interactions. With these variables, we can control for fluctuations in interaction patterns due to situational factors such as time of day. For example, the covariate entailing the number of interactions within the past 2 hr captures a tendency of individuals to repeatedly interact within a short (i.e., two-hour) time window. This can, for instance, be because the smartphone is placed somewhere where there are frequent signal interruptions (e.g., in a backpack) or because the person had many short interactions within the given time window (e.g., because they were attending a social mixer). This variable thus captures some unobserved heterogeneity between situations. Such time and window-control variables are commonly used when modeling interaction dynamics (Hoffman et al., 2019; Stadtfeld & Block, 2017).

We further control for the effect of short-term effects of student’s smartphone usage on social interactions by including a variable in the model that captures whether or not the student had been using the smartphone in the previous hour (value = 1) or not (value = 0).

We did not compute the “time in state” for the overnight observation; hence, the time gaps between the last measure in the evening and the first measure in the morning are not part of the analyses.

## Results

### Descriptives

#### Social interactions

In total 74,645 interactions were recorded. Participants were classified to be part of a social interaction on average 1512.62 times in total (*SD* = 541.26) and on average 27.28 times per day (*SD* = 14.50). On average, interactions lasted for 10.02 min (*SD* = 14.46). The average time between two interactions was 45.50 min (*SD* = 259.07).

#### Loneliness

Participants reported on average a level of 2.27 (*SD* = 0.71) of intimate loneliness, 1.54 (*SD* = 0.61) of relational loneliness, and 1.76 (*SD* = 0.66) of collective loneliness.

### RQ1: Is loneliness associated with the time between social interactions (i.e., interaction rate)?

Table 2 shows the results of the multistate analysis. The second to fourth columns in Table 2 show the estimates for the transitions from the alone state to the interaction state (i.e., the effect of interaction rate). Although all estimates with regards to loneliness were negative, indicating a lower tendency of individuals with higher loneliness scores to move from an alone state to an interaction state, none of the three loneliness subscales measured were significantly associated with the rate of joining social interactions. Other variables in the model, however, were important predictor variables: Students were more likely to engage in social interactions during the day, if they were in more interactions in the past 2 hr and 24 hr, and if they were in longer interactions within the past 2 hr. Students that were on their smartphones prior to the social interaction were less likely to engage in social interactions.

**Table 2.** Estimates of the multistate model for transitions interaction to alone states and alone to interaction states.

	Alone to interaction				Interaction to alone			
	Est.		*SE*	*p*-value	Est.		*SE*	*p*-value
Weekend (ref. weekday)	0.164		0.100	.102	0.078	**	0.027	.004
Morning (ref. night)	1.352	***	0.106	<.001	−0.324	***	0.043	<.001
Afternoon	1.289	***	0.100	<.001	−0.213	***	0.027	<.001
Evening	0.949	***	0.097	<.001	−0.187	***	0.031	<.001
Mean duration of interaction in past 2 hr [in min]	0.012	***	0.002	<.001	−0.024	***	0.001	<.001
Number of interactions in past 24 hr	0.012	***	0.003	<.001	<.001		0.001	.701
Number of interactions in past 2 hr	0.088	***	0.008	<.001	0.011	*	0.005	.027
phone used within last hour (ref. Not used)	−0.457	***	0.064	<.001	−0.116	***	0.022	<.001
Intimate loneliness T1	−0.019		0.095	.844	−0.044		0.049	.366
Relational loneliness T1	0.001		0.078	.987	0.099	**	0.036	.006
Collective loneliness T1	−0.060		0.088	.490	0.009		0.046	.848

*Note*. ${\text{ N}}_{\text{obersvations}}=74,645$ corresponding to $N_{transitions}=132,372$ of $N_{individuals}\;=\;45$. Cox and Snell pseudo- $R^{2}=18.9$, SE = Standard Error.

*p (two-sided) < .05, **p < .01, ***p < .001

### RQ2: Is loneliness associated with the duration of social interactions?

Columns five through seven in Table 2 show the estimates for the transitions from the interaction state to the alone state (i.e., the leaving rates or the interaction duration). In this sub-model, only relational loneliness measured was significantly associated with the rate of leaving social interactions. The effect of β=0.099 suggests that individuals with one unit higher in relational loneliness at T1 were exp(β)=1.10 times more likely to leave social interactions than those with a one-unit lower value in relational loneliness. In other words, those individuals at the high end of the relational loneliness scale (value = 4) are 35% (exp(3∗β)=1.33) more likely to leave an interaction at any given timepoint than an individual at the low end of the scale (value = 1), thus, the duration of their interactions was shorter. With regards to the covariates, students left social interactions more likely on weekends, less likely during the day, and less likely if they had longer interactions within the past 2 hr. Also, those who had more interactions within the past 2 hr were slightly more likely to leave social interactions. Being on the phone prior to the start of the social interaction was associated with a lower risk of leaving social interactions.

We additionally conducted several sensitivity analyses and a post-hoc power analysis – reported in the Supplementary Materials. The sensitivity analyses showed that the findings are robust when additionally controlling for extraversion, which had no effect on the joining rate or the leaving rate, $\beta_{joining}=\;-0.051,\;95\%CI\left[\;-0.182;\;0.082\right]$, $\beta_{leaving}=\;-0.015,\;95\%CI\;\left[-0.091;\;0.060\right]$. Additionally, we conducted an analysis with loneliness as a unidimensional construct, which had no effect on the joining or the leaving rate, $\beta_{joining}=\;-0.079,\;95\%CI\;\;\left[-0.235;0.077\right]$, $\beta_{leaving}\;=\;0.066,\;95\%CI\left[-0.025;\;0.156\right]$.

## Discussion

In this article, we demonstrated how fine-grained social interaction data collected through participant’s smartphones can be modeled with multistate survival models to provide new insights into the relationship between social interaction dynamics and psychological phenomena, such as loneliness. For this, we used a multi-method combination of novel, automatically sensed social interaction data and survey methods. Using data from a longitudinal ambulatory assessment study design, we investigated how types of loneliness (intimate, relational, collective) predict the rate (RQ1) and duration (RQ2) of social interactions.

Our analyses provide no suggestion that intimate and collective loneliness predict the time between social interactions (i.e., interaction rate) or the duration of social interactions. Relational loneliness was associated with leaving social interactions more quickly but not with joining them at a higher rate. In other words, we found evidence that relational loneliness is related to shorter social interactions but not necessarily to the rate of interactions.

Earlier research using self-reported interactions suggested that loneliness is associated with less time spent in interactions (Lee & Ko, 2017; Wheeler et al., 1983). In the present study, we cannot confirm this notion. Only for relational loneliness, we found that it may be related to fewer interactions. This could indicate that especially people who are relationally lonely are more withdrawn from social interactions (Watson & Nesdale, 2012). Relational loneliness reflects a feeling that one does not belong to a peer group. Many interactions in students' lives take part in group settings (Elmer & Stadtfeld, 2020). Thus, it is likely that especially those students who feel a lack of belonging are likely to leave such interactions more quickly. As earlier studies did not differentiate between different types of loneliness, it is not clear whether their results were also mainly driven by students experiencing relational loneliness. Yet, in a sensitivity analysis, we also examined loneliness as a unidimensional construct, comparable to previous studies. Here, we did not find significant associations with loneliness (see Supplementary Materials for details). Thus, the differences could also reflect a difference between self-reported and automatically detected interactions, showing the value of using different measures to examine such questions.

Beyond the substantive findings on the relationship between loneliness and social interactions, this article contributes to the literature on social-interaction research by demonstrating how social interactions can be studied using passive-sensing methods and multistate survival analyses. A particular advantage of passively sensing social interactions compared to survey-based measures is that social behavior can be measured in real-life settings without any input required from the participant, thus not interrupting the natural flow of day-to-day life (e.g., by filling out an ESM survey). As a result, specific measurement biases are reduced, as participants seem to be prone to miss reporting experience sampling surveys when in a social interaction (Sun et al., 2021).

Although passive-sensing and digital phenotyping are promising avenues for psychological science to study people unobtrusively in real life (Torous et al., 2016), their application still warrants more systematic validation studies (e.g., Elmer et al., 2019), data processing guidelines (Montag et al., 2020), and tailored analysis methods (Onnela, 2021).

A limitation of our empirical demonstration is that we examined a small, specific population of individuals (i.e., 45 male-majority computer science students). Hence, we cannot generalize our findings to other groups of populations. Future studies should examine the interplay between social interaction dynamics and loneliness in larger and more diverse populations.

Another limitation is that we only measured trait loneliness. It would be relevant to investigate the interplay between social interactions and state loneliness on a moment-to-moment basis. Ambulatory assessment methods using automated social interaction sensor technologies in combination with experience sampling methods – that allow assessing self-reports of state loneliness and qualitative characteristics of social interactions – thus hold great potential in the investigation of short-term (weekly, daily, momentary) loneliness and social interaction dynamics.

Finally, it would have been interesting to examine not only how loneliness predicts social interaction dynamics but also how social interaction dynamics predict *changes* in loneliness. Although in our study loneliness was also measured after the ambulatory assessment phase, the number of participants with valid loneliness change scores (*N* = 36) was very low, not yielding sufficient statistical power to study changes in loneliness. Using procedures to conduct analyses with small sample sizes (e.g., bootstrapping), we, nevertheless, report an exploratory analysis on how social interaction dynamics are associated with changes in loneliness in the Supplementary Materials. These exploratory analyses suggest that individuals who engage in longer interactions tend to report a decrease in collective loneliness (for a detailed discussion, see Section three in the Supplementary Materials).

## Conclusion

Drawing on smartphone sensors to study fine-grained social interaction dynamics in daily life settings can advance our understanding of people’s social life. This study advances knowledge on social interaction dynamics with a unique multi-method study design by exemplifying how the richness of sensor data extracted from individuals’ smartphones can be used to draw conclusions about the interrelations between social interaction dynamics and loneliness.

## Supplemental Material

Supplemental Material - Modeling social interaction dynamics measured with smartphone sensors: An ambulatory assessment study on social interactions and loneliness Click here for additional data file. Supplemental Material for Modeling social interaction dynamics measured with smartphone sensors: An ambulatory assessment study on social interactions and loneliness by Timon Elmer and Gerine Lodder in Journal of Social and Personal Relationships

## Note

1 It is important to note that being alone (i.e., solitude) and feeling lonely are two distinct constructs. Whereas solitue is the objective social state of being alone, loneliness describes the subjective feeling of a discepancy between the actual and desired social state (Peplau & Perlman, 1982).

## References

[bibr1-02654075221122069] Bachmann A. (2015). Towards smartphone-based sensing of social interaction for ambulatory assessment. [Paper presentation]. Proceedings of the 2015 ACM International Joint Conference on Pervasive and Ubiquitous Computing and Proceedings of the 2015 ACM International Symposium on Wearable Computers - UbiComp ’15, Umeda, Osaka, 9–11 September 2015 (pp. 423–428). 10.1145/2800835.2801642

[bibr2-02654075221122069] Bijwaard G. E. (2014). Multistate event history analysis with frailty. Demographic Research, 30(1), 1591–1620. 10.4054/DemRes.2014.30.58

[bibr3-02654075221122069] Cacioppo J. T. Hawkley L. C. (2009). Perceived social isolation and cognition. Trends in Cognitive Sciences, 13(10), 447–454. 10.1016/j.tics.2009.06.005 19726219 PMC2752489

[bibr4-02654075221122069] Cacioppo J. T. Hawkley L. C. Ernst J. M. Burleson M. Berntson G. G. Nouriani B. Spiegel D. (2006). Loneliness within a nomological net: An evolutionary perspective. Journal of Research in Personality, 40(6), 1054–1085. 10.1016/j.jrp.2005.11.007

[bibr5-02654075221122069] Cacioppo S. Grippo A. J. London S. Goossens L. Cacioppo J. T. (2015). Loneliness: Clinical import and interventions. Perspectives on Psychological Science, 10(2), 238–249. 10.1177/1745691615570616 25866548 PMC4391342

[bibr6-02654075221122069] Cox D. R. (1972). Regression models and life-tables. Journal of the Royal Statistical Society. Series B (Methodological), 34(2), 187–220. 10.1111/j.2517-6161.1972.tb00899.x

[bibr7-02654075221122069] De Jong Gierveld J. Van Tilburg T. G. Dykstra P. A. (2016). New ways of theorizing and conducting research in the field of loneliness and social isolation. In Vangelisti A. L. Perlman D. (Eds.), The Cambridge handbook of personal relationships (pp, 391–404). Cambridge University Press.

[bibr8-02654075221122069] Elmer T. Chaitanya K. Purwar P. Stadtfeld C. (2019). The validity of RFID badges measuring face-to-face interactions. Behavior Research Methods, 51(5), 1–19. 10.3758/s13428-018-1180-y 30997659 PMC6797650

[bibr9-02654075221122069] Elmer T. Stadtfeld C. (2020). Depressive symptoms are associated with social isolation in face-to-face interaction networks. Scientific Reports, 10(1), 1444. 10.1038/s41598-020-58297-9 31996728 PMC6989520

[bibr145-02654075221122069] Feese S. Tröster G. (2013). Robust voice activity detection for social sensing. In Proceedings of the 2013 ACM conference on Pervasive and ubiquitous computing adjunct publication (UbiComp ’13 Adjunct) (pp. 931-938). New York, NY, USA: Association for Computing Machinery. 10.1145/2494091.2497347

[bibr10-02654075221122069] Fulford D. Mote J. Gonzalez R. Abplanalp S. Zhang Y. Luckenbaugh J. Onnela J.-P. Busso C. Gard D. E. (2021). Smartphone sensing of social interactions in people with and without schizophrenia. Journal of Psychiatric Research, 137, 613-620. 10.1016/j.jpsychires.2020.11.002 33190842 PMC8084875

[bibr11-02654075221122069] Gochmann V. Ohly S. Kotte S. (2022). Diary studies, a double‐edged sword? An experimental exploration of possible distortions due to daily reporting of social interactions. Journal of Organizational Behavior. 10.1002/job.2633

[bibr12-02654075221122069] Goffman E. (1956). The presentation of self in everyday life. Anchor Books. 10.2307/2089106

[bibr13-02654075221122069] Goossens L. Beyers W. (2002). Comparing measures of childhood loneliness: Internal consistency and confirmatory factor Analysis. Journal of Clinical Child and Adolescent Psychology, 31(2), 252–262. 10.1207/S15374424JCCP3102_10 12056108

[bibr14-02654075221122069] Hawkley L. C. Cacioppo J. T. (2010). Loneliness matters: A theoretical and empirical review of consequences and mechanisms. Annals of Behavioral Medicine, 40(2), 218–227. 10.1007/s12160-010-9210-8 20652462 PMC3874845

[bibr15-02654075221122069] Hoffman M. Block P. Elmer T. Stadtfeld C. (2019). A model for the dynamics of face-to-face interactions in social groups. Network Science, 28(S1), S4–S25. 10.1017/nws.2020.3

[bibr16-02654075221122069] Holt-Lunstad J. Smith T. B. Baker M. Harris T. Stephenson D. (2015). Loneliness and social isolation as risk factors for mortality: A meta-analytic review. Perspectives on Psychological Science, 10(2), 227–237. 10.1177/1745691614568352 25910392

[bibr17-02654075221122069] John O. P. Srivastava S. (1999). The Big Five trait taxonomy: History, measurement, and theoretical perspectives. In L. A. Pervin & O. P. John (Eds.), Handbook of personality: Theory and research (2nd ed., pp. 102–138). New York: Guilford Press.

[bibr18-02654075221122069] Lane N. Mohammod M. Lin M. Yang X. Lu H. Ali S. Doryab A. Berke E. Choudhury T. Campbell A. (2011). BeWell: A smartphone application to monitor, model and promote wellbeing. PrevasiveHealth 2011.

[bibr19-02654075221122069] Lee Y. Ko Y. G. (2017). Feeling lonely when not socially isolated: Social isolation moderates the association between loneliness and daily social interaction. Journal of Social and Personal Relationships, 35(10), 1–16. 10.1177/0265407517712902

[bibr20-02654075221122069] Luhmann M. Bohn J. Holtmann J. Koch T. Eid M. (2016). I’m lonely, can’t you tell? Convergent validity of self- and informant ratings of loneliness. Journal of Research in Personality, 61(4), 50–60. 10.1016/j.jrp.2016.02.002

[bibr21-02654075221122069] Maes M. Qualter P. Vanhalst J. Van den Noortgate W. Goossens L. (2019). Gender differences in loneliness across the lifespan: A meta-analysis. European Journal of Personality, 33(6), 642–654. 10.1002/per.2220

[bibr22-02654075221122069] Mehl M. R. Pennebaker J. W. Crow D. M. Dabbs J. Price J. H. (2001). The electronically activated recorder (EAR): A device for sampling naturalistic daily activities and conversations. Behavior Research Methods, Instruments, & Computers, 33(4), 517–523. 10.3758/BF03195410 11816455

[bibr23-02654075221122069] Mehl M. R. Vazire S. Holleran S. E. Clark C. S. (2010). Eavesdropping on happiness: Well-being is related to having less small talk and more substantive conversations. Psychological Science, 21(4), 539–541. 10.1177/0956797610362675 20424097 PMC2861779

[bibr24-02654075221122069] Mendes J. P. M. Moura I. R. Van de Ven P. Viana D. Silva F. J. S. Coutinho L. R. Teixeira S. Rodrigues J. J. P. C. Teles A. S. (2022). Sensing apps and public data sets for digital phenotyping of mental health: Systematic review. Journal of Medical Internet Research, 24(2), e28735. 10.2196/28735 35175202 PMC8895287

[bibr25-02654075221122069] Milek A. Butler E. A. Tackman A. M. Kaplan D. M. Raison C. L. Sbarra D. A. Vazire S. Mehl M. R. (2018). Eavesdropping on happiness” revisited: A pooled, multisample replication of the association between life satisfaction and observed daily conversation quantity and quality. Psychological Science, 29(9), 1451–1462. 10.1177/0956797618774252 29969949 PMC6139582

[bibr26-02654075221122069] Miller G. (2012). The smartphone psychology manifesto. Perspectives on Psychological Science, 7(3), 221–237. 10.1177/1745691612441215 26168460

[bibr27-02654075221122069] Montag C. Sindermann C. Baumeister H. (2020). Digital phenotyping in psychological and medical sciences: A reflection about necessary prerequisites to reduce harm and increase benefits. Current Opinion in Psychology, 36(3), 19–24. 10.1016/j.copsyc.2020.03.013 32361334

[bibr28-02654075221122069] Onnela J.-P. (2021). Opportunities and challenges in the collection and analysis of digital phenotyping data. Neuropsychopharmacology, 46(1), 45–54. 10.1038/s41386-020-0771-3 32679583 PMC7688649

[bibr29-02654075221122069] Peplau L. A. Perlman D. (1982). Perspective on loneliness. In Peplau L. A. Perlman D. (Eds), Loneliness: A sourcebook of current theory, research and therapy. Wiley.

[bibr30-02654075221122069] Podsakoff P. M. MacKenzie S. B. Lee J. Y. Podsakoff N. P. (2003). Common method biases in behavioral research: A critical review of the literature and recommended remedies. Journal of Applied Psychology, 88(5), 879–903. 10.1037/0021-9010.88.5.879 14516251

[bibr31-02654075221122069] Putter H. Fiocco M. Geskur R. B. (2007). Tutorial in biostatistics: Competing risks and multi-state models. Statistics in Medicine, 26(11), 2389–2430. 10.1002/sim.2712 17031868

[bibr32-02654075221122069] Qualter P. Vanhalst J. Harris R. Van Roekel E. Lodder G. Bangee M. Maes M. Verhagen M. (2015). Loneliness across the life span. Perspectives on Psychological Science, 10(2), 250–264. 10.1177/1745691615568999 25910393

[bibr33-02654075221122069] Quoidbach J. Taquet M. Desseilles M. de Montjoye Y.-A. Gross J. J. (2019). Happiness and social behavior. Psychological Science, 30(8), 095679761984966. 10.1177/0956797619849666 31268832

[bibr34-02654075221122069] Rabbi M. Ali S. Choudhury T. Berke E. (2011). Passive and in-situ assessment of mental and physical well-being using mobile sensors. [Paper presentation]. Proceedings of the 13th International Conference on Ubiquitous Computing - UbiComp ’11, Beijing, China, 17–21 September 2011 (pp. 385–394). 10.1145/2030112.2030164 PMC4180507 25285324

[bibr35-02654075221122069] Reis H. T. Wheeler L. (1991). Studying social interaction with the rochester interaction record. Advances in Experimental Social Psychology, 24(C), 269–318. 10.1016/S0065-2601(08)60332-9

[bibr36-02654075221122069] Russell D. W. (1996). UCLA loneliness scale (version 3): Reliability, validity, and factor structure. Journal of Personality Assessment, 66(1), 20–40. 10.1207/s15327752jpa6601_2 8576833

[bibr37-02654075221122069] Schwartz-Mette R. A. Shankman J. Dueweke A. R. Borowski S. Rose A. J. (2020). Relations of friendship experiences with depressive symptoms and loneliness in childhood and adolescence: A meta-analytic review. Psychological Bulletin, 146(8), 664–700. 10.1037/bul0000239 32406698

[bibr38-02654075221122069] Stadtfeld C. Block P. (2017). Interactions, actors, and time: Dynamic network actor models for relational events. Sociological Science, 4(14), 318–352. 10.15195/v4.a14

[bibr39-02654075221122069] Stoolmiller M. Snyder J. (2006). Modeling heterogeneity in social interaction processes using multilevel survival analysis. Psychological Methods, 11(2), 164–177. 10.1037/1082-989X.11.2.164 16784336

[bibr40-02654075221122069] Sun J. Rhemtulla M. Vazire S. (2021). Eavesdropping on missing data: What are university students doing when they miss experience sampling reports? Personality & Social Psychology Bulletin, 47(11), 1535–1549. 10.1177/0146167220964639 33342369

[bibr41-02654075221122069] Torous J. Kiang M. V. Lorme J. Onnela J.-P. (2016). New tools for new research in psychiatry: A scalable and customizable platform to empower data driven smartphone research. JMIR Mental Health, 3(2), e5165. 10.2196/mental.5165 PMC4873624 27150677

[bibr42-02654075221122069] Trull T. J. Ebner-Priemer U. W. (2014). The role of ambulatory assessment in psychological science. Current Directions in Psychological Science, 23(6), 466–470. 10.1177/0963721414550706 25530686 PMC4269226

[bibr43-02654075221122069] van Roekel E. Ha T. Scholte R. H. J. Engels R. C. M. E. Verhagen M. (2016). Loneliness in the daily lives of young adults: Testing a socio-cognitive model. European Journal of Personality, 30(1), 19–30. 10.1002/per.2028

[bibr44-02654075221122069] Vogel N. Ram N. Conroy D. E. Pincus A. L. Gerstorf D. (2017). How the social ecology and social situation shape individuals’ affect valence and arousal. Emotion, 17(3), 509–527. 10.1037/emo0000244 27869467

[bibr45-02654075221122069] Wang R. Chen F. Chen Z. Li T. Harari G. Tignor S. Zhou X. Ben-Zeev D. Campbell A. T. (2014). Studentlife: Assessing mental health, academic performance and behavioral trends of college students using smartphones. Brush A. J. (Eds), UbiComp 2014 - Proceedings of the 2014 ACM International Joint Conference on Pervasive and Ubiquitous Computing (pp. 3–14). A.C.M., 2014. 10.1145/2632048.2632054

[bibr46-02654075221122069] Watson J. Nesdale D. (2012). Rejection sensitivity, social withdrawal, and loneliness in young adults. Journal of Applied Social Psychology, 42(8), 1984–2005. 10.1111/j.1559-1816.2012.00927.x

[bibr47-02654075221122069] Wheeler L. Reis H. Nezlek J. (1983). Loneliness, social interaction, and sex roles. Journal of Personality and Social Psychology, 45(4), 943–953. 10.1037/0022-3514.45.4.943 6631669

