# Supplementary material for: Modeling social interaction dynamics measured with smartphone sensors: An ambulatory assessment study on social interactions and loneliness
Source: J Soc Pers Relat. 2022 Aug 20;40(2):654–69. doi: 10.1177/02654075221122069 (PMC9941651; doi:10.1177/02654075221122069)
Supplement: Supplemental Material - Modeling social interaction dynamics measured with smartphone sensors: An ambulatory assessment study on social interactions and loneliness [file sj-pdf-1-spr-10.1177_02654075221122069.pdf]

Table of Content for the Supplementary Materials

Sensitivity Analyses 2
Power analysis 10
Exploring changes in loneliness 11

Sensitivity Analyses

We performed several sensitivity analyses (see Tables S1 to S6). First, we examined whether we would replicate results when we examined all loneliness subscales separately, rather than in a single analysis where they controlled for each other. Results were similar, indicating that relational loneliness was significantly associated with the duration of interactions ($\beta = 0.078, 95\% CI$ [0.010; 0.146]), and none of the other associations reached significance (for details, see Tables S1 to S3).

Second, we repeated both RQ1 and RQ2 for loneliness as a unidimensional construct by averaging a loneliness score across all 20 items of the UCLA loneliness scale (see Table S4). Regarding RQ1, there were no significant associations between T1 loneliness and the joining ($\beta = -0.079, 95\% CI$ [-0.235; 0.077]) or leaving of social interactions $\beta = 0.066, 95\% CI$ [-0.025; 0.156]).

Third, we additionally controlled for the role of extraversion in modeling social interaction dynamics (RQ1, RQ2). The results of a multistate model with the additional control variable for extraversion can be found in Table S5. The effects of extraversion on the rate to join or leave social interactions were not-significant, $\beta_{joining} = -0.051, 95\% CI$ [-0.182; 0.081], $\beta_{leaving} = -0.015, 95\% CI$ [-0.091; 0.060]. The associations of the three loneliness variables with the rate of joining or leaving social interactions did not change with regards to the direction, size, and significance level of the effect.

Table S1

Sensitivity analysis with estimates of the multistate model for transitions interaction to alone states and alone to interaction states with intimate loneliness

	alone to interaction				interaction to alone			
			95% CI				95% CI	
	Est.		lower	upper	Est.		lower	upper
Weekend (ref. weekday)	0.165		-0.039	0.370	0.081	**	0.029	0.133
Morning (ref. night)	1.360	***	1.151	1.570	-0.328	***	-0.409	-0.248
Afternoon	1.297	***	1.096	1.498	-0.207	***	-0.259	-0.155
Evening	0.937	***	0.743	1.130	-0.185	***	-0.248	-0.122
Mean duration of interaction in past 2h [in min]	0.012	***	0.009	0.015	-0.024	***	-0.027	-0.022
Number of interactions in past 24h	0.012	***	0.007	0.018	0.000		-0.002	0.002
Number of interactions in past 2h	0.092	***	0.076	0.108	0.011	*	0.001	0.021
Intimate loneliness T1	-0.057		-0.185	0.071	0.009		-0.067	0.086

Note. $\mathrm{N_{obersvations}} = 74{,}645$ corresponding to $N_{transitions} = 132{,}372$ of $\mathrm{N_{individuals}} = 45$.

Table S2

Sensitivity analysis with estimates of the multistate model for transitions interaction to alone states and alone to interaction states with relational loneliness

	alone to interaction				interaction to alone			
			95% CI				95% CI	
	Est.		lower	upper	Est.		lower	upper
Weekend (ref. weekday)	0.164		-0.042	0.370	0.081	**	0.028	0.134
Morning (ref. night)	1.358	***	1.147	1.570	-0.337	***	-0.416	-0.258
Afternoon	1.297	***	1.096	1.498	-0.213	***	-0.267	-0.158
Evening	0.938	***	0.746	1.130	-0.189	***	-0.252	-0.127
Mean duration of interaction in past 2h [in min]	0.012	***	0.008	0.015	-0.024	***	-0.027	-0.022
Number of interactions in past 24h	0.012	***	0.007	0.018	0.000		-0.002	0.002
Number of interactions in past 2h	0.092	***	0.076	0.108	0.012	*	0.002	0.021
Relational loneliness T1	-0.050		-0.217	0.116	0.078	*	0.010	0.146

Note. $\mathrm{N_{obersvations}} = 74{,}645$ corresponding to $N_{transitions} = 132{,}372$ of $\mathrm{N_{individuals}} = 45$.

Table S3

Sensitivity analysis with estimates of the multistate model for transitions interaction to alone states and alone to interaction states with collective loneliness

	alone to interaction				interaction to alone			
			95% CI				95% CI	
	Est.		lower	upper	Est.		lower	upper
Weekend (ref. weekday)	0.164		-0.040	0.368	0.081	**	0.029	0.133
Morning (ref. night)	1.361	***	1.150	1.573	-0.335	***	-0.415	-0.254
Afternoon	1.299	***	1.098	1.500	-0.210	***	-0.266	-0.154
Evening	0.939	***	0.745	1.133	-0.185	***	-0.250	-0.120
Mean duration of interaction in past 2h [in min]	0.012	***	0.008	0.015	-0.024	***	-0.027	-0.022
Number of interactions in past 24h	0.012	***	0.006	0.018	0.000		-0.002	0.002
Number of interactions in past 2h	0.092	***	0.076	0.108	0.011	*	0.001	0.021
Collective loneliness T1	-0.065		-0.194	0.065	0.036		-0.035	0.108

Note. $\mathrm{N}_{\mathrm{obersvations}} = 74{,}645$ corresponding to $N_{transitions} = 132{,}372$ of $\mathrm{N}_{\mathrm{individuals}} = 45$.

Table S4

Sensitivity analysis with estimates of the multistate model for transitions interaction to alone states and alone to interaction states with unidimensional loneliness

	alone to interaction				interaction to alone			
			95% CI				95% CI	
	Est.		lower	upper	Est.		lower	upper
Weekend (ref. weekday)	0.166		-0.039	0.370	0.081	**	0.029	0.134
Morning (ref. night)	1.361	***	1.150	1.571	-0.339	***	-0.419	-0.260
Afternoon	1.298	***	1.098	1.499	-0.213	***	-0.267	-0.159
Evening	0.941	***	0.749	1.133	-0.189	***	-0.253	-0.125
Mean duration of interaction in past 2h [in min]	0.012	***	0.009	0.015	-0.024	***	-0.027	-0.022
Number of interactions in past 24h	0.012	***	0.006	0.018	0.000		-0.002	0.002
Number of interactions in past 2h	0.092	***	0.076	0.108	0.011	*	0.002	0.021
Loneliness T1	-0.083		-0.250	0.084	0.065		-0.026	0.156

Note. $\mathrm{N}_{\mathrm{obersvations}} = 74{,}645$ corresponding to $N_{transitions} = 132{,}372$ of $\mathrm{N}_{\mathrm{individuals}} = 45$.

Table S5

Sensitivity analysis with estimates of the multistate model for transitions interaction to alone states and alone to interaction states controlling for extraversion

	alone to interaction				interaction to alone			
	Est.		SE	p-value	Est.		SE	p-value
Weekend (ref. weekday)	0.222	*	0.095	0.019	0.076	**	0.026	0.004
Morning (ref. night)	1.385	***	0.106	0.000	-0.352	***	0.046	0.000
Afternoon	1.313	***	0.106	0.000	-0.223	***	0.030	0.000
Evening	1.013	***	0.096	0.000	-0.207	***	0.030	0.000
Mean duration of interaction in past 2h [in min]	0.011	***	0.002	0.000	-0.025	***	0.001	0.000
Number of interactions in past 24h	0.013	***	0.003	0.000	0.000		0.001	0.886
Number of interactions in past 2h	0.087	***	0.008	0.000	0.009		0.005	0.056
phone used within last hour (ref. Not used)	-0.450	***	0.059	0.000	-0.123	***	0.023	0.000
Extraversion	-0.051		0.067	0.448	-0.015		0.038	0.694
Intimate loneliness T1	-0.032		0.104	0.757	-0.061		0.054	0.259
Relational loneliness T1	0.022		0.081	0.790	0.081	*	0.035	0.021
Collective loneliness T1	-0.075		0.096	0.434	0.019		0.046	0.673

Note. $\mathrm{N}_{\mathrm{obersvations}} = 74{,}645$ corresponding to $N_{transitions} = 132{,}372$ of $\mathrm{N}_{\mathrm{individuals}} = 45$.

Table S6

Sensitivity analysis with estimates of the multistate model for transitions interaction to alone states and alone to interaction states with additional squared loneliness variables

	alone to interaction				interaction to alone			
	Est.		SE	p-value	Est.		SE	p-value
Weekend (ref. weekday)	0.161		0.102	0.115	0.082	**	0.028	0.003
Morning (ref. night)	1.347	***	0.106	0.000	-0.323	***	0.044	0.000
Afternoon	1.290	***	0.099	0.000	-0.212	***	0.028	0.000
Evening	0.952	***	0.097	0.000	-0.186	***	0.030	0.000
Mean duration of interaction in past 2h [in min]	0.012	***	0.002	0.000	-0.024	***	0.001	0.000
Number of interactions in past 24h	0.012	***	0.003	0.000	0.000		0.001	0.738
Number of interactions in past 2h	0.088	***	0.008	0.000	0.011	*	0.005	0.028
phone used within last hour (ref. Not used)	-0.453	***	0.062	0.000	-0.119	***	0.022	0.000
Intimate loneliness T1	0.241		0.469	0.607	0.158		0.244	0.518
Relational loneliness T1	-0.251		0.525	0.633	0.529	*	0.240	0.028
Collective loneliness T1	0.087		0.332	0.795	-0.308		0.196	0.115

Intimate loneliness T1 squared	-0.055	0.106	0.604	-0.045	0.053	0.401
Relational loneliness T1 squared	0.072	0.149	0.632	-0.115	0.067	0.086
Collective loneliness T1 squared	-0.034	0.085	0.689	0.083	0.046	0.071

Note. $\mathrm{N}_{\mathrm{obersvations}} = 74{,}645$ corresponding to $N_{transitions} = 132{,}372$ of $\mathrm{N}_{\mathrm{individuals}} = 45$.

Power analysis

We further conducted a post-hoc power analysis to examine the statistical power of this multistate analysis. The power analysis showed that with $\alpha = .05, 1 - \beta = .80, N_{individuals} = 45$ and a total of $n \geq 72{,}000$ interaction observations, effect sizes of Cohen's d = *.50* (medium effect size, corresponding to a Person correlation of r = .24) can be detected. For details on the power analysis see Figure S1 or the respective R-script on OSF.

Figure S1. Power analysis for the multistate analysis

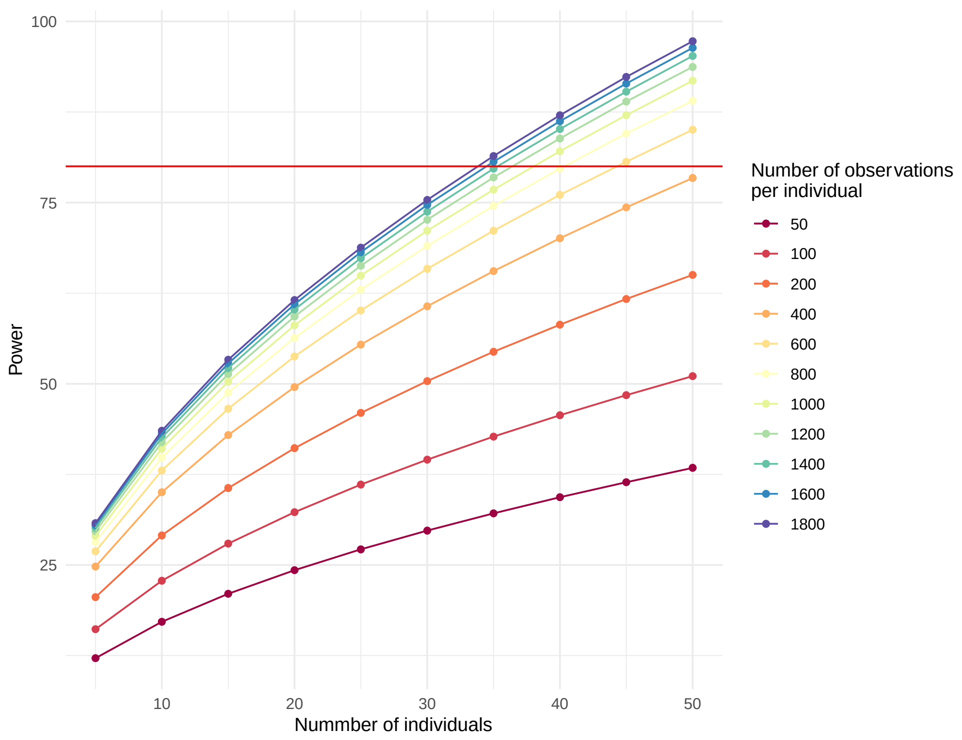

Exploring changes in loneliness

So far, studies have only assessed how loneliness measured prior to social interaction assessment predicted the (self-reported) quantity of social interactions at a later point in time (Wheeler et al., 1983). However, little is known about how the social interactions experienced in daily life are associated with *changes* in loneliness, using a longitudinal study design. In line with the RAM model (Qualter et al., 2015), more frequent social interactions or social interactions that are longer in duration may reduce feelings of loneliness, as they may signal a sense of reconnection. At the same time, in line with the loneliness model by Cacioppo and colleagues (2006; 2009; 2010), cognitive biases may cause lonely individuals to interpret their interactions negatively, and as such lonely individuals may not benefit from having many or long interactions – in contrast, this may even be a risk continued loneliness for those individuals who interpret their social environment negatively. Indeed, studies employing a momentary assessment design indicated that being company reduced state loneliness for some but not all adolescents (Van Roekel et al., 2014). It remains unclear whether the frequency or duration of interactions will reduce feelings of loneliness. In an exploratory analysis, we therefore examine a second Research Question 3 (RQ3): Are characteristics of day-to-day social interactions (i.e., frequency and duration) associated with changes in loneliness?

Methods. For our exploratory analyses on predicting loneliness change (RQ3), we will use only those 36 participants that filled out the pre-ambulatory assessment (T1) *and* the post-ambulatory assessment (T2) survey. Because the sample of students who participated in the T1 and T2 survey was rather small (N = 36), inferential statistical analyses would not have enough statistical power to detect significant differences. In a power analysis, with $\alpha = .05$ (type I error) and $\beta = .20$ (type II error), a sample size of N = 36 could only detect correlations above $r = .45$ (Hulley et al., 2013). However,

just because sample size is small and the design of this study is underpowered, does not mean that descriptive statistical analyses cannot provide insights into the nature of the phenomena (Siebert & Siebert, 2009). By relying on a measure of within-person change from T1 to T2 loneliness, the variability in scores due to between-person variability is reduced (i.e., there is less noise in the data). As a result of the decreased noise, not as many datapoints are needed to detect meaningful statistical associations (Tambling & Anderson, 2014). Furthermore, statistical techniques such as bootstrapping can aid in making correlation estimates of analyses with small samples sizes more reliable (Tambling & Anderson, 2014). By resampling data from an observed distribution, bootstrapping methods allow researchers to draw more accurate inferences about population parameters (Efron & Tibshirani, 1986). Instead of inferential statistical analyses (such as regression models), we thus explore associations between changes in loneliness and characteristics of social interactions with scatterplots and discuss the size and confidence intervals of bootstrapped correlation coefficients.

For this we compute changes in the three types of loneliness (intimate, relational, collective) from before (T1) to after (T2) the ambulatory assessment phase ($\Delta L = L_{T2} - L_{T1}$). Variables characterizing social interaction dynamics consist of (a) the number of social interactions measured during the ambulatory assessment phase, and (b) the mean duration of these social interactions. These measures of social interaction characteristics have been shown to be important predictors of loneliness and related mental health measures (Elmer et al., 2020; Wheeler et al., 1983).

Results. Table S1 shows the mean and *SD* values of T1 and T2 loneliness as well as loneliness change. An increase in loneliness between T1 and T2 was observed for 67% of the individuals in intimate loneliness, 83% in relational loneliness, and 69% in collective loneliness. Hence, although the average across all individuals in loneliness change was close to zero, it seems that

for most individuals, loneliness got slightly higher, and for only a few loneliness got a lot lower. For details, see the descriptive Figure S2.

We also examined whether changes in unidimensional loneliness (i.e., the average of all items on the scale combined) were related to interaction dynamics. The descriptive associations with the frequency or duration of interactions were close to zero (frequency: $r(34) = .09, 95\%CI\ [-.28; .55]$, duration: $r(34) = .00, 95\%CI[-.36; .43]$).

Table S1. *Mean and Standard Deviation of loneliness variables at T1, T2 and their change.*

	**Type of loneliness**	**Mean**	**SD**
T1	Collective	1.76	0.66
	Relational	1.54	0.61
	Intimate	2.27	0.71
T2	Collective	1.82	0.74
	Relational	1.63	0.71
	Intimate	2.27	0.65
Change (T2-T1)	Collective	0.06	0.46
	Relational	0.07	0.43
	Intimate	-0.06	0.55

Figure S1

Individual-level trajectories of loneliness subscales from T1 to T2. Each individual is represented by a separate color.

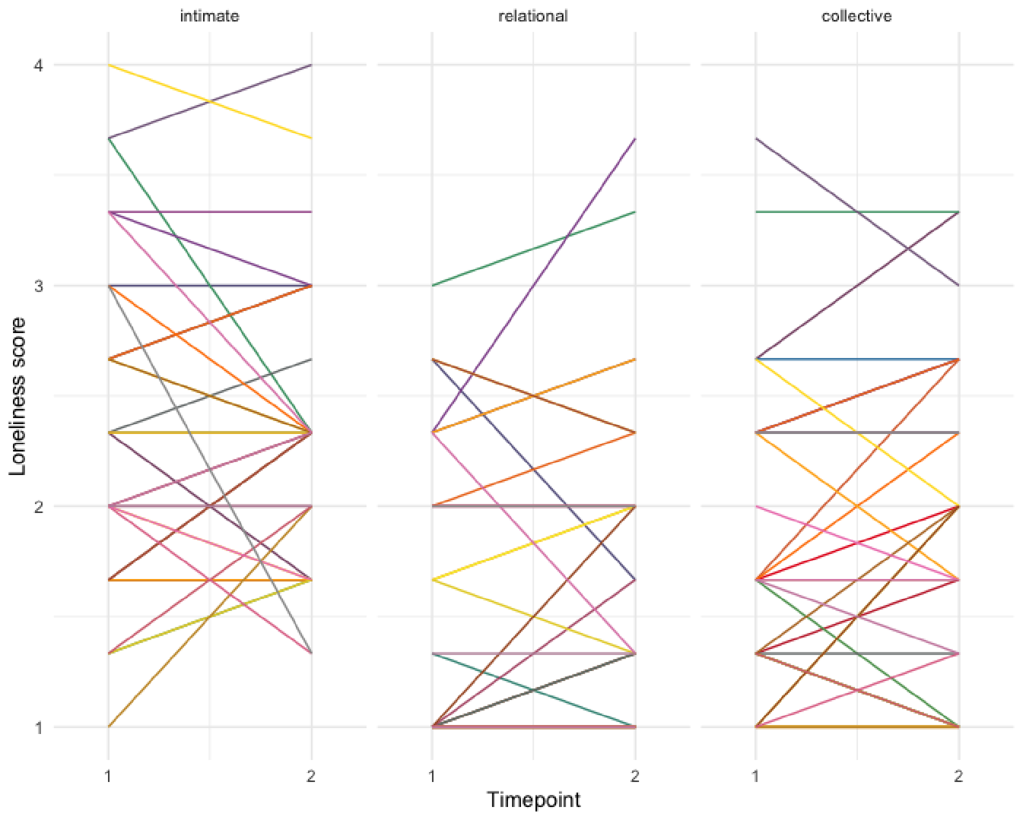

Figure S2 shows the bivariate scatterplots between all variable combinations and a fitted line. Most noteworthy is the association between the mean duration of social interactions and change in collective loneliness. This association was estimated to be $r(34) = -.30, 95\% CI\ [-.02; -.61]$ in a bootstrapped correlation analysis (with 10,000 samples), indicating that having longer social interactions is associated with a decrease in collective loneliness. None of the other bivariate associations relating to our research question reached significance, and most were close to zero. There was a trend for an association between relational loneliness ($r(34) = -.13, 95\% CI\ [-.58; .29]$) and

intimate loneliness ($r(34) = -.10, 95\%CI\ [-.42; .20]$) and the frequency of interactions, but the effects were relatively small and did not reach significance. All correlation coefficients and associated 95% Confidence Intervals are reported in Table S8.

Figure S2. *Scatterplots (each point is one individual's observation) and fitted bivariate associations (line) of loneliness and social interaction variables.*

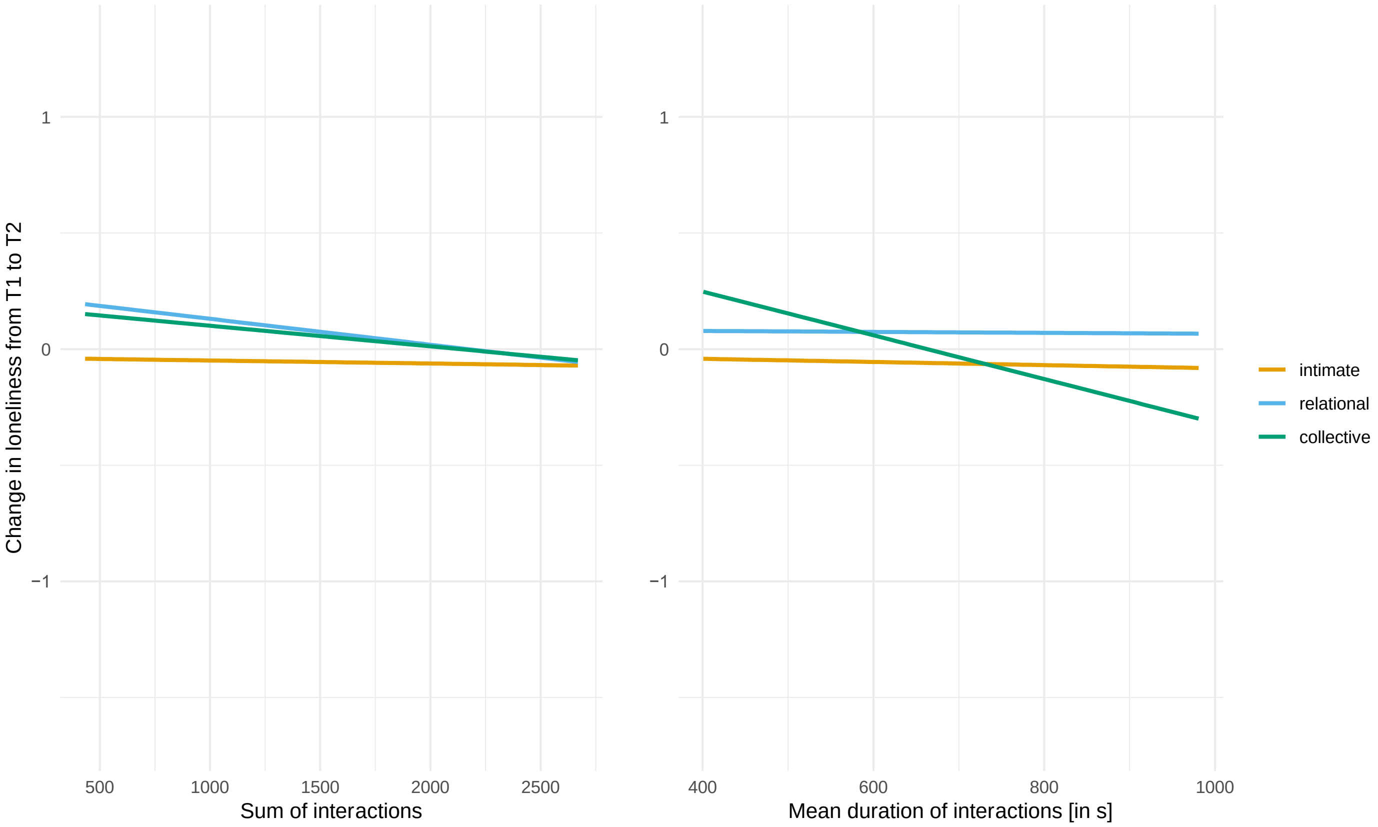

Discussion of exploratory analysis

Due to the small sample size, we investigated changes in loneliness (RQ3) only with descriptive analysis methods in an exploratory analysis. Hence, these findings need to be interpreted with caution,

as the statistical power for these analyses was low and only descriptive bivariate associations were tested. Using bootstrapping correlation methods, we observed that an increase in collective loneliness was associated with shorter social interactions during the ambulatory assessment phase, but not with the frequency of interactions. Collective loneliness reflects deficits in the collective space, the personal space consisting of others that share your social identity (S. Cacioppo et al., 2015). Early adulthood is characterized by consolidation of one’s identity, which partly happens through social interactions (Madsen & Collins, 2018). Those who have shorter interactions may be less likely to discover others whom they share a social identity with, thereby resulting in increased feelings of collective loneliness. Yet, as this study was explorative in its nature and the sample size was small, future research should be conducted to confirm these ideas.

Indeed, the size of the sample was one of the main limitations of the present study, which limited our options to examine our second research question, regarding the association between change in loneliness and the structure of interactions. Studies with small sample sizes but intensive longitudinal design strategies (i.e., many observations per individual – such as in our study) are becoming increasingly common because of their usefulness for hypotheses generation and idiographic analyses (Zuidersma et al., 2020). Nevertheless, future studies should include larger samples when aiming to predict person-level outcomes such as loneliness.

Despite the underpowered nature of the analysis, this exploratory analysis contribute to the literature on social sensor data in mental health research (e.g., Elmer & Stadtfeld, 2020; Wang et al., 2014), by showcasing how smartphone-sensed interaction data are associated with changes in loneliness.

Table S8

Bootstrapped Pearson correlation coefficient and 95% Confidence Interval bounds between social interaction variables and change in loneliness from T1 to T2.

		Pearson correlation coefficient	95% CI Lower bound	95% CI Upper bound
Sum of interactions	*loneliness intimate change*	*-0.01*	*-0.34*	*0.32*
	loneliness relational change	*-0.14*	*-0.58*	*0.28*
	loneliness collective change	*-0.10*	*-0.42*	*0.20*
	loneliness change (full scale)	*0.09*	*-0.28*	*0.55*
	Mean duration of interactions	*0.01*	*-0.35*	*0.35*
Mean duration of interactions	*loneliness intimate change*	*-0.02*	*-0.34*	*0.29*
	loneliness relational change	*-0.01*	*-0.33*	*0.34*
	loneliness collective change	*-0.30*	*-0.60*	*-0.02*
	loneliness change (full scale)	*0.01*	*-0.35*	*0.43*
	Sum of interactions	*0.01*	*-0.35*	*0.35*

References

Cacioppo, J. T., & Hawkley, L. C. (2009). Perceived social isolation and cognition. *Trends in Cognitive Sciences*, *13*(10), 447–454. https://doi.org/10.1016/j.tics.2009.06.005

Cacioppo, J. T., Hawkley, L. C., Ernst, J. M., Burleson, M., Berntson, G. G., Nouriani, B., & Spiegel, D. (2006). Loneliness within a nomological net: An evolutionary perspective. *Journal of Research in Personality*, *40*(6), 1054–1085. https://doi.org/10.1016/j.jrp.2005.11.007

Cacioppo, S., Grippo, A. J., London, S., Goossens, L., & Cacioppo, J. T. (2015). Loneliness: Clinical Import and Interventions. *Perspectives on Psychological Science*, *10*(2), 238–249. https://doi.org/10.1177/1745691615570616

Efron, B., & Tibshirani, R. (1986). Bootstrap Methods for Standard Errors, Confidence Intervals, and Other Measures of Statistical Accuracy. *Https://Doi.Org/10.1214/Ss/1177013815*, *1*(1), 54–75. https://doi.org/10.1214/SS/1177013815

Elmer, T., Geschwind, N., Peeters, F., Wichers, M., & Bringmann, L. (2020). Getting Stuck in Social Isolation: Solitude Inertia and Depressive Symptoms. *Journal of Abnormal Psychology*, *129*(7), 713–723. https://doi.org/10.1037/abn0000588

Elmer, T., & Stadtfeld, C. (2020). Depressive symptoms are associated with social isolation in face-to-face interaction networks. *Scientific Reports*, 1–12. https://doi.org/10.1038/s41598-020-58297-9

Hawkley, L. C., & Cacioppo, J. T. (2010). Loneliness Matters: A Theoretical and Empirical Review of Consequences and Mechanisms. *Annals of Behavioral Medicine*, *40*(2), 218–227. https://doi.org/10.1007/s12160-010-9210-8

Hulley, S., Cummings, S., Browner, W., Grady, D., & Newman, T. (2013). *Designing clinical research: An epidemiologic approach*. Lippincott Williams & Wilkins.

Madsen, S. D., & Collins, W. A. (2018). Personal Relationships in Adolescence and Early Adulthood. In *The Cambridge Handbook of Personal Relationships* (pp. 135–147). Cambridge University Press. https://doi.org/10.1017/9781316417867.012

Qualter, P., Vanhalst, J., Harris, R., Van Roekel, E., Lodder, G., Bangee, M., Maes, M., & Verhagen, M. (2015). Loneliness Across the Life Span. *Perspectives on Psychological Science, 10*(2), 250–264. https://doi.org/10.1177/1745691615568999

Siebert, C., & Siebert, D. C. (2009). *Data Analysis with Small Samples and Non-Normal Data* (Vol. 148). Oxford University Press.

Tambling, R. B., & Anderson, S. R. (2014). Statistical analysis with small samples. *Advanced Methods in Family Therapy Research: A Focus on Validity and Change, January*, 401–419. https://doi.org/10.4324/9780203084526

Van Roekel, E., Goossens, L., Verhagen, M., Wouters, S., Engels, R. C. M. E., & Scholte, R. H. J. (2014). Loneliness, affect, and adolescents' appraisals of company: An experience sampling method study. *Journal of Research on Adolescence*, *24*(2), 350–363. https://doi.org/10.1111/jora.12061

Wang, R., Chen, F., Chen, Z., Li, T., Harari, G., Tignor, S., Zhou, X., Ben-Zeev, D., & Campbell, A. T. (2014). Studentlife: Assessing mental health, academic performance and behavioral trends of college students using smartphones. *UbiComp 2014 - Proceedings of the 2014 ACM International Joint Conference on Pervasive and Ubiquitous Computing*, 3–14. https://doi.org/10.1145/2632048.2632054

Wheeler, L., Reis, H., & Nezlek, J. (1983). Loneliness, Social Interaction, and Sex Roles. *Journal of Personality and Social Psychology*, *45*, 943–953.

Zuidersma, M., Riese, H., Snippe, E., Booij, S. H., Wichers, M., & Bos, E. H. (2020). Single-Subject Research in Psychiatry: Facts and Fictions. *Frontiers in Psychiatry*, *11*(November). https://doi.org/10.3389/fpsyt.2020.539777
